# Entomotherapeutic Role of *Periplaneta americana* Extract in Alleviating Aluminum Oxide Nanoparticles-Induced Testicular Oxidative Impairment in Migratory Locusts (*Locusta migratoria*) as an Ecotoxicological Model

**DOI:** 10.3390/antiox12030653

**Published:** 2023-03-06

**Authors:** Esraa A. Arafat, Doaa S. El-Sayed, Hussein K. Hussein, Justin Flaven-Pouchon, Bernard Moussian, Lamia M. El-Samad, Abeer El Wakil, Mohamed A. Hassan

**Affiliations:** 1 Department of Zoology, Faculty of Science, Alexandria University, Alexandria 21321, Egypt; 2 Chemistry Department, Faculty of Science, Alexandria University, Alexandria 21321, Egypt; 3 Interfaculty Institute for Cell Biology, Eberhard-Karls Universität Tübingen, 37073 Tübingen, Germany; 4 Université Côte d’Azur, Parc Valrose, 06000 Nice, France; 5 Department of Biological and Geological Sciences, Faculty of Education, Alexandria University, Alexandria 21526, Egypt; 6 Protein Research Department, Genetic Engineering and Biotechnology Research Institute (GEBRI), City of Scientific Research and Technological Applications (SRTA-City), New Borg El-Arab City 21934, Egypt; 7 University Medical Center Göttingen, Georg-August-University, 37073 Göttingen, Germany

**Keywords:** aluminum oxide NPs, *Locusta migratoria*, oxidative stress, testicular oxidative damages, *Periplaneta americana* extract-derived insects, antioxidant activity

## Abstract

In this study, we shed light for the first time on the usage of migratory locusts (*Locusta migratoria*) as an insect model to investigate the nanotoxicological influence of aluminum oxide (Al_2_O_3_) nanoparticles at low doses on testes, and evaluate the capacity of a whole-body extract of American cockroaches (*Periplaneta americana*) (PAE) to attenuate Al_2_O_3_ NPs-induced toxicity. Energy dispersive X-ray microanalyzer (EDX) analysis verified the bioaccumulation of Al in testicular tissues due to its liberation from Al_2_O_3_ NPs, implying their penetration into the blood–testis barrier. Remarkably, toxicity with Al engendered disorders of antioxidant and stress biomarkers associated with substantial DNA damage and cell apoptosis. Furthermore, histopathological and ultrastructural analyses manifested significant aberrations in the testicular tissues from the group exposed to Al_2_O_3_ NPs, indicating the overproduction of reactive oxygen species (ROS). Molecular docking analysis emphasized the antioxidant capacity of some compounds derived from PAE. Thus, pretreatment with PAE counteracted the detrimental effects of Al in the testes, revealing antioxidant properties and thwarting DNA impairment and cell apoptosis. Moreover, histological and ultrastructural examinations revealed no anomalies in the testes. Overall, these findings substantiate the potential applications of PAE in preventing the testicular impairment of *L. migratoria* and the conceivable utilization of locusts for nanotoxicology studies.

## 1. Introduction

Nanoparticles (NPs) are particles with at least one dimension less than 100 nm that have been identified as key elements of nanomaterials, which could be further modified for implementation in various sectors in terms of nanotechnology [1]. NPs are classified into different categories following their morphological features, size, and chemical characteristics. Furthermore, NPs can be put into predominant groups based on their physical and chemical properties, such as metal, ceramic, carbon-based, lipid-based, and polymeric NPs [2]. Given their outstanding and versatile properties, NPs, particularly metal NPs, have been receiving immense attention for applications in multiple fields, including industrial, environmental, biomedical, and electronic sectors [2,3]. Along with this extensive growth and revolution, the exposure of living organisms to these metal NPs is inevitable due to their high discharge into the ecological system, raising critical questions about their potential deleterious consequences [4]. Recent reports have launched discussions about the toxicological impacts of metal NPs, such as Al_2_O_3_ NPs and Ag NPs, on the reproductive system, demonstrating some evidence about their conceivable mechanisms, which have not been fully comprehended [5,6,7]. Among these metal NPs, Al_2_O_3_ NPs are one of the most widely utilized, and account for about 20% of the global nanoparticle market, since they could be exploited in coating, textile functionalization, and medicine for drug delivery application, and are substantially implemented as aluminum-based adjuvants in vaccines to modulate the immune response while boosting human immunization [8,9]. Previous studies have demonstrated that the accumulation of Al_2_O_3_ NPs inside biological tissues could disturb biological pathways, inducing critical toxicological episodes [10,11].

This is principally due to the discharging of Al from the nanoparticles within the respective tissues, generating surpluses of reactive oxygen species (ROS) and mutations of DNA, which provoke the dysfunction of cells and the substantive disruption of the antioxidant defense system and other metabolic mechanisms [5,11]. These detrimental events could be easily instigated due to the infiltration of Al_2_O_3_ NPs throughout different tissues, including the blood–testis barrier in terms of the reproductive system, inciting oxidative stress alongside its complications, which breaks up cellular homeostasis and further leads to the impairment of testicular tissues [10]. The study of the harmful effects of metal NPs on the reproductive system using insects as bioindicators has recently come to light in order to evaluate environmental pollution and comprehend critical biological problems [12,13]. Recently, migratory locusts, *Locusta migratoria* (Linnaeus, 1758), have received a lot of attention for their use in environmental toxicity assessments because they have inherent advantages, such as a short life cycles and non-specific living demands [12,14,15]. Significantly, the immune system of *L. migratoria* comprises humoral-based and cell-mediated immune reactions, including the synergy of hemocytes, fat bodies, and circulating hemolymph peptides for the elimination or deactivation of xenobiotics [15]. Furthermore, multiple actions could be performed by insects to counteract oxidative damage, including enzymatic and non-enzymatic antioxidants; for instance, carboxylesterase (CarE), and glutathione S-transferase (GST) are important detoxifying enzymes in insects. Moreover, they can metabolize a large number of toxins to maintain their physiological activities [16].

To hinder the toxicological effects, in particular the oxidative stress engendered by Al_2_O_3_ NPs, many endeavors have been dedicated to discovering natural products with intrinsic antioxidant capacities. The major studies focused on antioxidant-derived plants that contain bioactive polyphenolic compounds such as quercetin to reduce oxidative damage in hepatic cells after treatment with Al_2_O_3_ NPs [17,18,19]. So far, very little attention has been paid to antioxidant-derived insects to tackle the deleterious consequences of the metal NPs. Traditional Chinese medicine has widely used an insect-derived whole-body extract of the cockroach *Periplaneta americana* [20]. It has been reported that it has remarkable antimicrobial [21,22] and anti-inflammatory properties [23].

Even though there is a lot of attention given to appraising the toxicological influences of nanoparticles in relation to testicular tissues, no reports to date have comprehensively probed the deleterious effects of Al_2_O_3_ NPs at low doses. Moreover, to the best of our knowledge, no studies have evaluated the prophylactic activity of *Periplaneta americana* extract (PAE) against any types of metal NPs in order to thwart various disturbances of biological pathways within the testicular tissues of *L. migratoria*. Herein, we extensively study the deleterious effects of Al_2_O_3_ NPs in the testicular tissues of locusts through evaluating physiological characteristics, DNA impairment, and cell apoptosis. Furthermore, the histopathological and ultrastructural features of the testicular sections of locusts subjected to Al_2_O_3_ NPs were inspected. On the other hand, comparable tests were conducted to assess the efficacy of PAE as a protective extract for male locusts before being exposed to the Al_2_O_3_ NPs to counteract the harmful effects caused by the nanoparticles.

## 2. Materials and Methods

### 2.1. Collection of Insects

Adult migratory locusts, *L. migratoria* (Linnaeus, 1758) (Orthoptera, Acrididae), were collected from an organic field cultured with corn in Giza governorate, Egypt, before being identified and housed in the Entomology laboratory at the Faculty of Science, Alexandria University, Egypt, in standard wood cages with ten individuals in each, of which 50% were males. They were maintained under standard conditions (temperature: 29.4 ± 3.5 °C; photoperiod: light:dark 12:12 h; humidity: 46.5 ± 9.4%) with unlimited access to water and food.

### 2.2. Al_2_O_3_ NPs Characterization

To probe the morphology and particle size of Al_2_O_3_ NPs (Nanotech Egypt for Photo-Electronics, Giza, Egypt), three samples were examined by a scanning electron microscope (SEM, JEOL JSM-5300, Tokyo, Japan) at an accelerating voltage of 20 kV and a transmission electron microscope (TEM, JEM-1400 Plus, Tokyo, Japan) at an acceleration voltage of 80 kV. Fourier transform infrared spectroscopy (FT-IR, Shimadzu 8400S, Kyoto, Japan) was also used to characterize the Al_2_O_3_ NPs.

### 2.3. Preparation of Al_2_O_3_ NPs Suspension and PAE Solution

To prepare a stock suspension of Al_2_O_3_ NPs, 1 mg/mL Al_2_O_3_ NPs were suspended in saline solution, followed by sonication using a Branson 450 sonicator (Branson Ultrasonics Crop, Danbury, CT, USA). The working solution was then prepared, and the final volume of each dose was adapted to the mass of each insect. PAE (Citeq Biologics, Groningen, Netherlands) stock solution was prepared by dissolving in a 0.9% NaCl solution according to the manufacturer’s instructions. 

### 2.4. Experimental Design

Prior to commencing the experiments, the weights of male migratory locusts were estimated at an average of 1.03 g in order to adapt the injection dose. To determine the dose of Al_2_O_3_ NPs for treating the migratory locusts, six groups of the migratory locusts were randomly established (10 insects/group) and designated from Al-G1 to Al-G6; these were injected with different doses at final concentrations of 0.01, 0.02, 0.03, 0.04, 0.05, and 0.06 mg/g body weight, respectively. On the other hand, to evaluate the toxicity of PAE and select the safest dose for locusts, three groups of insects (10 insects/group) termed PAE1, PAE2, and PAE3 were injected with PAE at doses of 0.01, 0.03, and 0.05 mg/g body weight, respectively. The control groups of insects in both investigations were injected with saline solution. The locusts were monitored for 10 days, and the mortality and behavior of insects were perceived and reported on a daily basis. According to mortality and behavior evaluations of male locusts, 60 locusts were randomly divided into three groups (20 insects/group): the control group, which was injected with a saline solution; the second group was injected with a single dose of Al_2_O_3_ NPs (0.03 mg/g body weight), and the third group was injected with a single dose of PAE (0.05 mg/g body weight) before being injected after 24 h with a dose of Al_2_O_3_ NPs (0.03 mg/g body weight). The injections were carried out in the intersegmental membrane between the 3rd and 4th abdominal sternites by means of a 0.5 mL BD hypodermic syringe (27 gauge, ½ inch needle).

### 2.5. Dissection Procedures

Adult locusts were cooled for 10 min at 4 °C. The testicular tissues were dissected from adult male locusts of the three groups mentioned above on ice under the microscope after being injected with 0.02 mL of 4% formaldehyde:1% glutaraldehyde (4F:1G) buffer (pH 7.2) in the abdominal region as the fixative solution, comprising 10 mL of formaldehyde (40%), 4 mL of glutaraldehyde (25%), 1.16 g of monobasic sodium phosphate, and 0.27 g of NaOH, completed to 100 mL by Milli-Q water as previously described [24], for scanning electron microscope–energy dispersive X-ray microanalyzer (SEM-EDX), histological, and ultrastructural analyses. The collected testicular tissues used for biochemical assays were preserved at −80 °C until use.

### 2.6. X-ray Detection of Al in the Testes of L. migratoria

To assess the levels of Al accumulated in the testicular tissues of adult *L. migratoria*, three samples from each group were examined by means of SEM (Jeol JSM-5300, Tokyo, Japan) linked with a Link-Isis EDX at an accelerating voltage of 20 kV. The identity of each peak was figured out automatically using the EDX software on the basis of the intensity of each element in comparison to reference elements.

### 2.7. Biochemical Evaluations

#### 2.7.1. Determination of Protein Content in Hemolymph and Total Hemocyte Count

Hemolymph and hemocyte isolations were performed using a modified protocol from Bergin et al. [25]. Adult locusts were anesthetized by cooling for 10 min at 4 °C. Afterward, hemolymph was collected by making a small puncture under the hind leg in the abdominal region [26]. The hemolymph was collected into heparin tubes and pooled (0.5 mL) from four locusts for hemocyte counting, with the remainder was pooled in a cold Eppendorf tube (0.5 mL) for total protein analysis. The collected hemolymph was diluted in 1:20 ratio with acidified physiological saline. The acidified physiological saline was prepared by mixing acetic acid with saline solution at a ratio of 0.1:2%. Hemocytes were separated from hemolymph by centrifugation at 1500× *g* for 5 min at 4 °C, washed twice, and then resuspended in a cold acidified physiological saline. The total hemocyte count was assessed using a hemocytometer by counting three independent samples from each group. Moreover, the total protein content of the hemolymph was evaluated using a commercial total protein kit (Spinreact, Girona, Spain).

#### 2.7.2. Biochemical Assays in Testicular Tissues

The dissected testicular tissues were homogenized in a phosphate buffer (pH 7.0) using a homogenizer (Kimble® 885300-0002, Sigma-Aldrich Chemie GmbH, Maryland, USA) then centrifuged at 16,100× *g* at 4 °C for 20 min, and the supernatant was then collected for biochemical assays.

The total proteins of testicular homogenates (mg/mg tissue) were assessed as described by Lowry et al. [27]. To appraise the lipid peroxidation, the malondialdehyde (MDA) level was determined following the protocol of Ohkawa et al. [28]. The assay is based on a reaction between 2-thiobarbituric acid (TBA) and MDA at 95 °C, resulting in the production of 2-thiobarbituric acid-reactive substance (TBARS) with a pink color, which was measured at 532 nm using a spectrophotometer. A reaction mixture free of a testis sample served as a control.

Alanine transferase (ALT) and aspartate transferase (AST) activities were quantified in the testicular homogenates utilizing BioSystems assay kits (Madrid, Spain), according to the manufacturer’s protocols. Furthermore, the performance of creatine kinase (CK) was assayed using a kit purchased from Spinreact Co. (Girona, Spain) following the manufacturer’s protocols.

To determine the activities of detoxifying enzymes, the activity of β-carboxylesterase (CarE) in the testicular tissue homogenates was measured following the protocol described by Thompson [29], and the activity of glutathione S-transferase (GST) was estimated according to the method reported by Carmagnol et al. [30].

Furthermore, the total antioxidant capacity (TAC) was evaluated using an assay kit supplied by Abcam Company (ab65329, Abcam Co., Berlin, Germany), while the activity of superoxide dismutase (SOD) was estimated utilizing the Elite^TM^ SOD Activity Assay Kit (MBS433565, MyBioSource Co., San Jose, CA, USA) following the manufacturer’s procedures. Additionally, glutathione peroxidase (GPx) activity was assessed following the method of Flohé and Günzler [31] and the activity of catalase (CAT) was determined following the procedures reported by Aebi [32].

To assess the index of inflammation in the testicular homogenates, the level of nitric oxide (NO) was assayed by a nitric oxide assay kit (ab65328, Abcam Co., Berlin, Germany) according to the manufacturer’s instructions.

#### 2.7.3. Molecular Docking and Computational Studies

Based on the findings of a previous study [20], some PAE compounds were chosen for molecular docking analysis in order to predict the antioxidant capacity implicated in our studied extract. The compounds, 7a-Methyl-1,2,3,6,7,7a-hexahydro-5H-inden-5-one (MHI), Methyl 4,8,12-trimethyltridecanoate (MTTD), Methylenebis (2,4,6triisopropylphenylphosphine) (MTP), Propanoic acid-ethyl ester (PAE) and oleic acid- methyl ester (OAM) were subjected to molecular docking analysis using two crystal macromolecular structures and 1AR5 and 8CAT targets to probe the antioxidant activity of these ligands. In the current study, a molecular docking simulation analysis was accomplished using Autodock software (version 4.2) (accessed on 1 December 2022) with the preparation of binding sites, then several energetic conformations’ production. The Discovery Studio program (https://www.3ds.com/products-services/biovia/) (accessed on 1 December 2022) was used for some visualization. The crystal macromolecular target proteins used in this study were downloaded from the protein data bank (https://www.rcsb.org/) (accessed on 1 December 2022). Grid parameters were located with box dimensions 60 × 44 × 56 Ǻ^3^ for 1AR5 and 62 × 54 × 60 Ǻ^3^ for 8CAT; the specified spaces included a large number of active macromolecular amino acid sites for best conformation prediction.

#### 2.7.4. Assessment of HSP70 and HSP90 mRNA Expressions

Three testicular tissues of *L. migratoria* were randomly selected for total RNA isolation utilizing the TRIzol™ Plus RNA Purification Kit (Invitrogen, USA) following the manufacturer’s instructions. The integrity and purity of the isolated RNA were evaluated by means of agarose gel electrophoresis and a spectrophotometer at 260/280 nm, respectively. The relative expression levels of HSP70 and HSP90 in testicular tissues of locusts were analyzed using a one-step RT-PCR reaction. The primers used in the RT-qPCR reactions were (Forward) 5′-AAA ATG AAA GAA ACG GCA GAG G-3′ and (Reverse) 5′-TAA TAC GCA GCA CAT TGA GAC C-3′ for HSP70, (Forward) 5′-GAT ACA TCC ACA ATG GGC TAC A-3′ and (Reverse) 5′-CTT GTC ATT CTT GTC CGC TTC A-3′ for HSP90, and (Forward) 5′-AAT TAC CAT TGG TAA CGA GCG ATT-3′ and (Reverse) 5′-TGC TTC CAT ACC CAG GAA TGA-3′ for housekeeping gene β-actin [33,34]. The RT-qPCR reactions were conducted using the Qiagen Rotor-Gene SYBR Green PCR Kit (QIAGEN, Hilden, Germany) in a 25 μL mixture containing 1 μg of RNA, 12.5 μL of SYBR Green, 2.5 μL of each primer and 9 μL of H_2_O. The RT-qPCR program consisted of an initial step at 95 °C for 5 min, followed by 40 cycles of 95 °C for 15 s and 60 °C for 10 s. The assays were performed by means of the Rotor-Gene Q using Rotor-Gene Q-Pure Detection version 2.1.0 (Qiagen, Montgomery, MD, USA). The quantification of the transcript levels of HSP70 and HSP90 mRNA was accomplished in accordance with the comparative 2-ΔΔCT method [35].

### 2.8. Cells Viability Assessment by Flow Cytometry

Flow cytometric analysis of testicular tissues was carried out using the TACS^TM^ Annexin-V-FITC apoptosis detection kit (TA4638, Germany) following the manufacturer’s instructions. Briefly, the cell suspension was obtained from the testicular tissues by homogenizing the tissues in cold phosphate-buffered saline (PBS, pH 7.4) at 4 °C. The cells were harvested and washed twice in PBS before being resuspended in 195 μL of binding buffer. Following this, 5 μL of Annexin-V-FITC conjugate reagent was added to the cell suspensions prior to being maintained in the dark for 10 min. After washing the cells, they were resuspended in 190 μL of binding buffer, followed by the addition of 10 μL propidium iodide solution. The states of different cells were then ascertained employing flow cytometry (Becton Dickinson, Franklin Lakes, NJ, USA) and the findings were assessed by means of Cell Quest Pro software version 5.2.1, 2005 (Becton Dickinson, San Jose, CA, USA).

### 2.9. Assessment of DNA Damage

The genotoxicity of the cells obtained from the testicular tissues of adult *L. migratoria* was evaluated by means of the comet assay according to Tice et al. [36]. The tissues were chopped using small dissecting scissors before being homogenized in a chilled buffer consisting of 0.075 M NaCl and 0.024 M Na_2_EDTA. The cell suspension was centrifuged at 700× *g* and 4 °C for 10 min, washed twice using the same buffer, and the cell pellet was then obtained. After that, cells were mixed with molten low-melting point (LM) agarose, followed by the spreading of the mixture over a frosted slide. The slides were immersed in lysis solution for 60 min before commencing the electrophoresis at a high pH (pH 13). Next, the slides were immersed in a neutralization buffer for 15 min. Afterward, the samples were dried, stained with ethidium bromide, and viewed by a Leitz Orthoplan epi-fluorescent microscope equipped with an excitation filter of 515–560 nm and a barrier filter of 590 nm. The microscope was connected to a computer-based image analysis system (Comet Assay V software, Perspective Instruments). To determine comet cells, 50 to 100 randomly selected cells per slide were used, and DNA damage was evaluated as tail length, % tail DNA, and tail moment accordingly.

### 2.10. Scanning Electron Microscope (SEM) Analysis of Testes

The testicular tissue samples were prepared for surveying by SEM as previously described [4]. Briefly, testicular tissues from each group were fixed in cold 4F:1G, which was prepared as described above (Section 2.5) in 0.1 M phosphate buffer solution (pH 7.2) for 3 h, followed by postfixation in 2% osmium tetroxide for 2 h at 4 °C. The samples were washed in PBS for 2 h at 4 °C, dehydrated in an increasing series of ethanol concentrations for 15 min each, and sectioned to 0.06 mm before being mounted on an aluminum stub. The samples were coated with gold palladium in a sputter-coating unit (JFC-1100 E), followed by inspection by means of an SEM.

### 2.11. Histological and Ultrastructural Analyses of Testicular Tissues

To examine the histological differences between the different groups of locusts, the testicular tissues were fixed, post-fixed, and dehydrated through the ethanol series as mentioned above in the SEM analysis. Subsequently, specimens were immersed in an Epon–Araldite mixture, and the tissues were sectioned (0.5 μm thick) by means of an LKB ultramicrotome (LKB Bromma, 2088 Ultrotome, Mississippi, USA) before being stained with toluidine blue. The slides were investigated employing a light microscope (Olympus CX31, Tokyo, Japan).

For the ultrastructural investigations, the prepared testicular tissues were sectioned (60 nm thick) on an LKB ultramicrotome and then picked up on 200 mesh naked copper grids. Following this, the testicular sections were stained with uranyl acetate and lead citrate before being scanned employing a transmission electron microscope (TEM, JEM-1400 Plus, Tokyo, Japan) at an acceleration voltage of 80 kV.

### 2.12. Statistical Analysis

All investigations were accomplished with 3–5 replicates for statistical analysis. To evaluate the significant differences in the results, statistical analyses of the raw data were carried out by means of GraphPad Prism (Version 8, GraphPad Software Inc., San Diego, CA, USA). For mortality analysis, the log-rank (Mantel-Cox) test was performed, and the Chi square was calculated. As regards the biochemical results, the normal (Gaussian) distribution of the data was assessed using the Shapiro–Wilk test, followed by one-way analysis of variance (ANOVA) with Tukey’s analysis for multiple comparisons between groups. All results are represented as mean ± SD, and they were considered significant at *p* ≤ 0.05.

## 3. Results

### 3.1. Characterization of Al_2_O_3_ NPs

**Figure 1:**
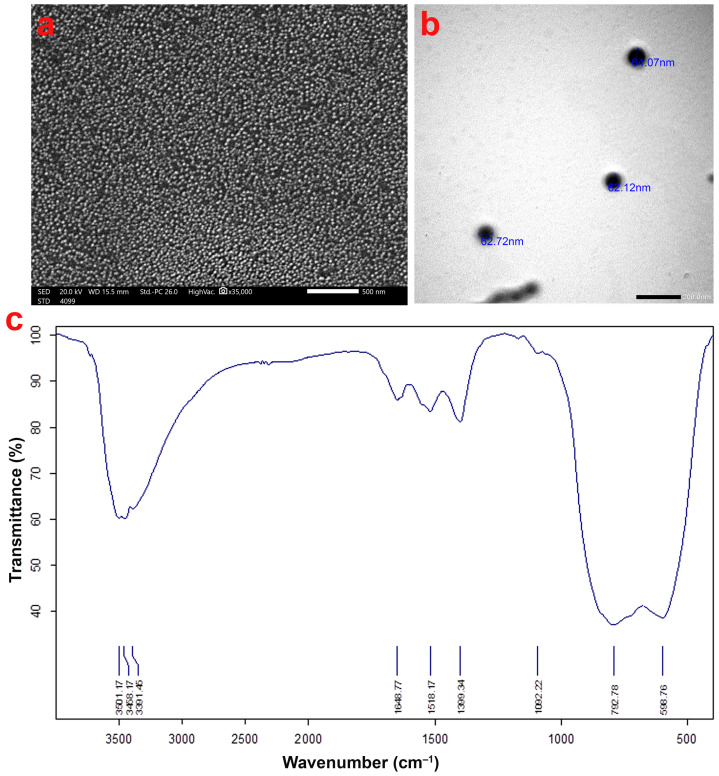
Characterization of Al_2_O_3_ NPs via SEM images (**a**), TEM images (**b**), and an FT-IR spectrum (**c**), validating the size of the nanoparticles used in this study, which is in agreement with the certificate provided by the manufacturer.

We have characterized the Al_2_O_3_ NPs used in this study by SEM, TEM, and FT-IR analyses, as depicted in Figure 1. It is apparent from the SEM and TEM images that Al_2_O_3_ NPs are spherical in nature with an average particle size of 62 nm, which correlates with the data supplied by the manufacturer. The results reveal that Al_2_O_3_ NPs less than 100 nm should be considered nanomaterials in accordance with the International Organization for Standardization (ISO).

Figure 1c delineates the FT-IR spectra of Al_2_O_3_ NPs, showing peaks below 1000 cm^−1^, which are distinct features of the studied nanoparticles. We also observed vibration peaks at 598 and 792 cm^−1^, which are attributed to Al–O stretching. Furthermore, the peaks at 3501, 3458, and 3391 cm^−1^ are assigned to the O–H stretching of the hydroxyl group. Additionally, the emergence of the peak at 1399 cm^−1^ is ascribed to Al=O, while the peak at 1092 cm^−1^ could be imputed to AL–O–H.

### 3.2. Survival and Mortality Analyses of L. migratoria Male after Injection with Al_2_O_3_ NPs and PAE

**Figure 2:**
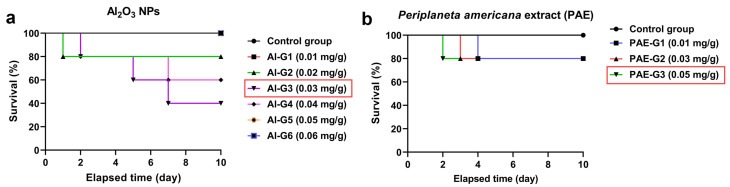
Mortality analyses for different groups of male locusts after injection with a single dose of either (**a**) Al_2_O_3_ NPs or (**b**) PAE in a dose-dependent manner after monitoring for 10 days. For the Al_2_O_3_ NPs treatment, six groups of male locusts along with the control group (10 insects/group) were established and injected with the nanoparticles at different doses of 0.01, 0.02, 0.03, 0.04, 0.05, and 0.06 mg/g body weight, respectively, and the Chi square was 12.43 with a *p* < 0.05. For PAE, three groups of male locusts along with the control group (10 insects/group) were established and injected with the extract at different concentrations of 0.01, 0.03, and 0.05 mg/g body weight, respectively, and the Chi square was 1.090 with a non-significant difference between groups. The red rectangle points to the doses selected to treat the male locusts for further investigations in the case of Al_2_O_3_ NPs and PAE.

The survival rates of male locusts after treatment with a single dose of Al_2_O_3_ NPs were appraised by monitoring six groups named Al-G1 to Al-G6 for ten days after administration with different concentrations of the nanoparticles. The Kaplan–Meier survival analysis was used to decipher the survival probability assays, as shown in Figure 2a, exposing significant differences between the insects injected with nanoparticles at concentrations of 0.03 and 0.04 mg/g body weight in comparison to the control locusts. Nevertheless, the group injected with 0.03 mg/g body weight of Al_2_O_3_ exhibited the highest mortality rate compared to the other groups of locusts, implying that this dose penetrated the most tissues and blood barriers, provoking deleterious impacts on the locust and possibly leading to death. Thus, we determined a dose of 0.03 mg/g body weight of Al_2_O_3_ NPs as a deleterious dose with regard to *L. migratoria*, for further investigations. On the other hand, we tested the toxicity of the PAE toward male locusts using three groups labeled from PAE-G1 to PAE-G3, which were injected with varying amounts of the extract, alongside the control group to determine the safe dose that could be used to alleviate the detrimental influences of Al_2_O_3_ NPs. It could be extrapolated from the data in Figure 2b, following the Kaplan–Meier survival analysis for PAE, that the results exhibited no significant differences in the mortality rate between various concentrations of the extract compared to the control insects. Therefore, we determined the highest concentration (0.05 mg/g body weight) of the extract as the potential protective dose in this work.

### 3.3. Evaluation of Al Accumulated in Testicular Tissues of Locusts

**Figure 3:**
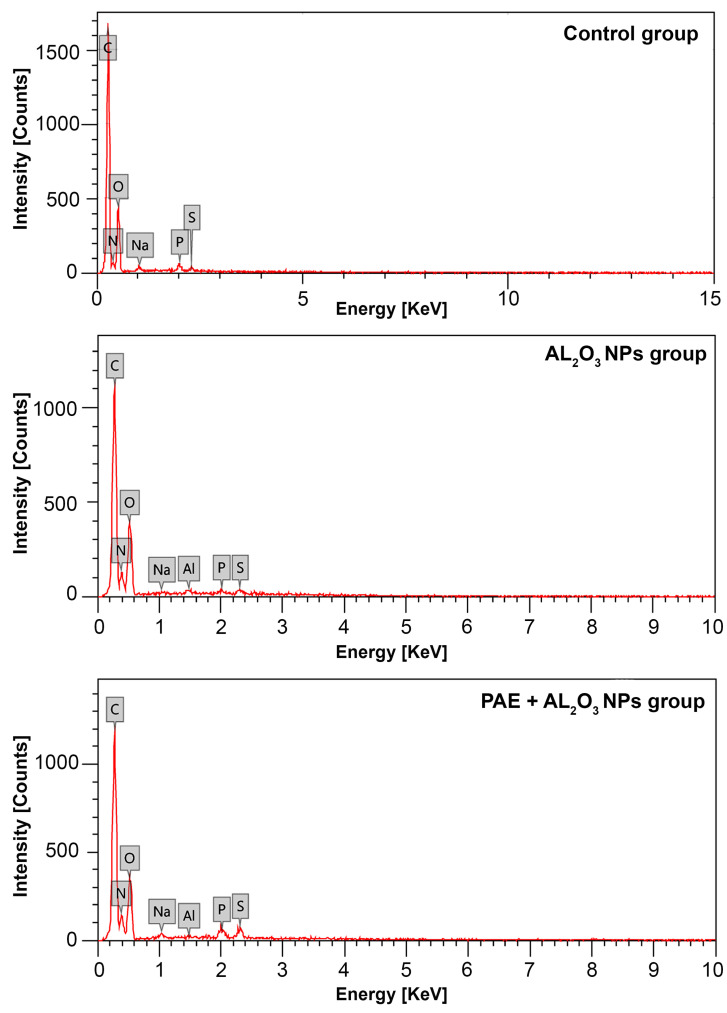
EDX analysis of testicular tissues harvested from control locusts, Al_2_O_3_ NPs-treated locusts, and a group of locusts treated with PAE + Al_2_O_3_ NPs, revealing an accumulation of Al in the Al_2_O_3_ NPs-treated groups, with different concentrations of Al in both treated groups.

The testicular tissues of male locusts dissected from the group treated only with Al_2_O_3_ NPs and the group co-treated with Al_2_O_3_ NPs and PAE were examined by EDX to evaluate the accumulation of Al compared to the control group as shown in Figure 3. Considering the EDX analysis of testicular tissues harvested from the control locusts, different peaks could be detectable, which are correlated with carbon (C), nitrogen (N), oxygen (O), sodium (Na), phosphorus (P), and sulfur (S). On the other hand, the EDX for the group of locusts treated only with Al_2_O_3_ NPs exhibited the substantial agglomeration of Al, reporting 0.25 ± 0.04% alongside comparable elements observed in the control insects with different amounts, as presented in Table 1, which could be elucidated by the anticipated physiological disturbance as a consequence of the exposure to Al_2_O_3_. By contrast, the locust group pretreated with PAE as a protective dose before being exposed to the Al_2_O_3_ NPs revealed a significant decrease in the level of Al accumulated in the testes, recording 0.07 ± 0.03%. Moreover, similar elements could be perceived in the latest group with concentrations close to the control group, except for sulfur, which could be estimated at high concentrations. This is most likely related to the sulfur contents of the PAE extract, since the most antioxidant and bioactive compounds possess sulfur, endowing them with favorable bioactivity.

### 3.4. Impact of Al_2_O_3_ NPs and Combinatorial Treatment of Al_2_O_3_ NPs and PAE on the Physiological Properties of L. migratoria

**Figure 4:**
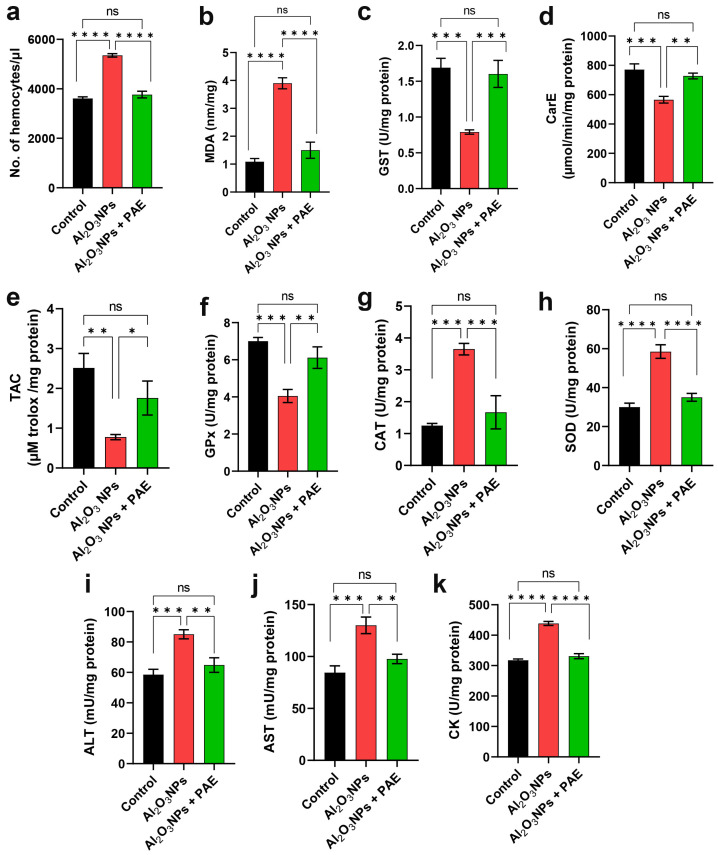
Biochemical analyses of testicular tissues after Al_2_O_3_ NPs injection in *L. migratoria* with or without PAE treatment. (**a**) Hemocytes total count, (**b**) malondialdehyde quantification by mg of testicular tissue, (**c**) glutathione S-transferase, (**d**) activity of the carboxylesterase E, (**e**) total antioxidant capacity, (**f**) glutathione peroxidase activity, (**g**) catalase activity, (**h**) superoxide dismutase level, (**i**) alanine transferase activity, (**j**) aspartate transferase activity, and (**k**) creatine kinase activity. All investigations were replicated at least three times and data are shown as mean ± SD. Data were analyzed with one-way ANOVA, followed by Tukey’s test for multiple comparisons. Different letters indicate significant differences between groups (**** *p* < 0.0001, *** *p* < 0.001, ** *p* < 0.01, * *p* < 0.05, and (ns) indicates a non-significant difference).

The density of hemocytes in the hemolymph was surveyed in the different experimental groups, as portrayed in Figure 4a. The total hemocyte count (THC) of the control insects was determined to be 3613 ± 65 hemocytes/µL. Compared to the control locusts, the THC significantly increased in the Al_2_O_3_ NPs-treated insects, reporting 5350 ± 70 hemocytes/µL. Noticeably, locusts that received combinatorial treatment with PAE (0.05 mg/g body weight), followed by Al_2_O_3_ NPs (0.03 mg/g body weight), exhibited a remarkable diminution (3767 ± 134 hemocytes/µL) in hemocyte density compared to those doped only with Al_2_O_3_ NPs. These findings suggest that injection with Al_2_O_3_ NPs induced inflammatory responses in *L. migratoria* that could be markedly mitigated by pretreatment with PAE.

In comparison to control locusts, the MDA levels were significantly promoted (Figure 4b), while the GST and CarE were remarkably diminished with regard to the testes of Al_2_O_3_ NPs-injected insects, as given in Figure 4c,d. Furthermore, considerable reductions in the TAC and GPx activities were observed in Al_2_O_3_ NPs-treated insects (Figure 4e,f), whereas the activities of CAT and SOD were significantly increased compared with the control insects (Figure 4g,h). Moreover, the activities of metabolic enzymes, including ALT, AST, and CK, were significantly enhanced in Al_2_O_3_ NPs-treated locusts compared to those of the control group (Figure 4i,j,k). Locusts pre-administered with a prophylactic dose of PAE, followed by Al_2_O_3_ NPs, manifested significant restoration of most of the studied biomarkers compared to insects receiving Al_2_O_3_ NPs alone. Taken together, these findings point out that the oxidative stress and toxicity induced by Al_2_O_3_ NPs could be significantly alleviated by PAE pretreatment.

**Figure 5:**
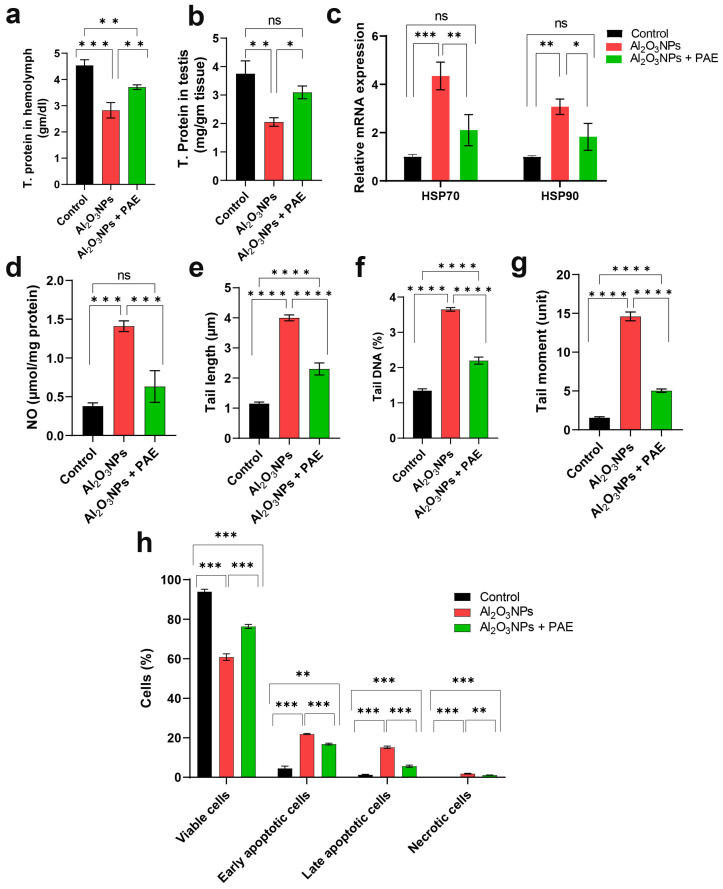
Total protein concentrations in (**a**) hemolymph and (**b**) testis, (**c**) relative expressions of HSP70 and HSP90 mRNA, and (**d**) nitric oxide were evaluated to determine the harmful effects induced in the testis by Al_2_O_3_ NPs injection in *L. migratoria* with or without PAE treatment. (**e**) Comet assay of DNA in cells extracted from testes, (**f**) tail length, DNA percentage in the comet tail, (**g**) tail moment, and (**h**) Annexin-V/PI assay results were also assessed. All investigations were replicated at least three times and data are shown as mean ± SD. For the comet assay, 50 to 100 randomly selected cells per slide were used. Data were analyzed with one-way ANOVA, followed by Tukey’s test for multiple comparisons. Different letters indicate significant differences between groups (**** *p* < 0.0001, *** *p* < 0.001, ** *p* < 0.01, * *p* < 0.05, and (ns) indicates a non-significant difference).

From the data in Figure 5a, it is evident that the single application of Al_2_O_3_ NPs (0.03 mg/g body weight) in male locusts provoked a noticeable lessening in the total protein content in both testicular tissues and the hemolymph compared to the control insects (Figure 5a). In contrast, the pre-exposure of male locusts to PAE improved the protein content of the hemolymph. Moreover, the pre-treatment of male locusts with PAE ameliorated the total protein contents in the testis, with no significant difference compared to the control locusts, as shown in Figure 5b.

To delve into the expression of important stress factors, we investigated the impacts of Al_2_O_3_ NPs on the expression of HSP 70 and HSP 90 in the locust testes, as depicted in Figure 5c. The results reveal a significant upregulation in both HSP 70 and HSP 90 genes in Al_2_O_3_ NPs-treated locusts compared to control insects. By contrast, the locust group co-treated with PAE + Al_2_O_3_ NPs demonstrated significant downregulations of both genes as a result of pre-treatment with PAE. These results clearly indicate that PAE could modulate the Al_2_O_3_ NPs-induced stress response, which supports the previous biochemical assays.

Besides this, the NO level in the testicular tissues of the Al_2_O_3_ NPs-injected locusts was significantly raised in comparison with the control group, whereas the pre-exposure of locusts to PAE prior to being doped with Al_2_O_3_ NPs significantly lowered this effect by 45%, as illustrated in Figure 5d. The reduction in NO levels in the testes of locusts treated with PAE emphasizes the antioxidant and anti-inflammatory properties of the PAE, implying that the PAE compounds may have the capacity to scavenge nitric oxide.

### 3.5. Evaluation of DNA Impairment by Comet Assay

The biochemical findings clearly point out that several metabolic pathways were affected by Al_2_O_3_ NPs. In addition, they reveal that the pretreatment with PAE could be remarkably effective in counteracting the adverse effects of Al_2_O_3_ NPs, restoring the major metabolic activities of the cells. We thus investigated the capacity of the PAE to reinstate the dominant functions of cells alongside the impact of exposure to Al_2_O_3_ NPs in the testicular tissues of locusts on the DNA integrity of the testicular cells. To assess DNA integrity in testicular tissues of *L. migratoria* after Al_2_O_3_ NPs intoxication, we conducted a comet assay (Figure 5e–g). Three parameters were estimated: percentage of DNA in comet tail, length of comet tail, and tail moment. As anticipated, the administration of Al_2_O_3_ NPs instigated genotoxic consequences in the testes of *L. migratoria*. Specifically, the level of DNA impairment was substantially higher in the testicular tissues of locusts that were only treated with the Al_2_O_3_ NPs, compared to both the control and PAE + Al_2_O_3_ NPs groups. Besides this, the amount of DNA in the comet tail was noticeably higher in the testicular tissues of the group exposed only to Al_2_O_3_ NPs, compared to the control and the PAE + Al_2_O_3_ NPs locusts. Moreover, the mean DNA percentage in the comet tail of the testicular tissue of the Al_2_O_3_ NPs-injected locusts was about three times higher than in the control ones, while PAE treatment resulted in a significant reduction of 40% compared to the Al_2_O_3_ NPs-injected locusts. Collectively, these outcomes show a prominent protective influence of PAE against Al_2_O_3_ NPs-induced genotoxicity, verifying our biochemical analyses.

### 3.6. Assessment of Cell Viability by Flow Cytometric Analysis

**Figure 6:**
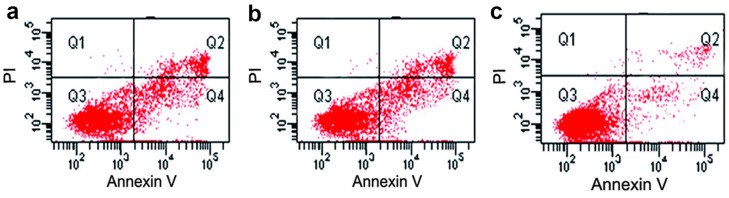
Flow cytometric analyses utilizing Annexin-V-FITC of (**a**) control locusts, (**b**) Al_2_O_3_ NPs-treated locusts, and (**c**) locusts treated with PAE + Al_2_O_3_ NPs. Q1, Q2, Q3, and Q4 signify necrotic cells, late apoptotic cells, viable cells, and early apoptotic cells, respectively.

The Annexin-V-FITC assay has shown that a single application of Al_2_O_3_ NPs provoked a substantial disturbance of live, dead, and apoptotic cells in the testicular tissues of migratory locusts, as illustrated in Figure 5h and Figure 6. Evidently, we discerned a significant reduction of 40% in living cells, associated with remarkable rises in apoptotic (early and late) cells in the Al_2_O_3_ NPs-injected locusts compared to the control insects. In contrast to these findings, PAE treatment counteracted this effect by increasing viable cells and decreasing apoptotic cells. These findings along with those obtained from the comet analysis demonstrate the effectiveness of PAE as a counteractive dose to rescue cell viability and their ideal characteristics in *L. migratoria* testicular cells.

### 3.7. Molecular Docking Analysis

**Figure 7:**
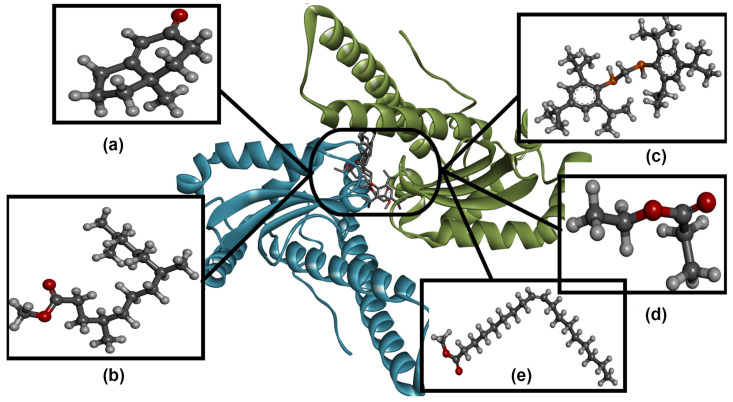
Docking pose scores of the studeid ligands (**a**) MHI, (**b**) MTTP, (**c**) MTP, (**d**) PAE and (**e**) OAM with target protein 1AR5.

**Figure 8:**
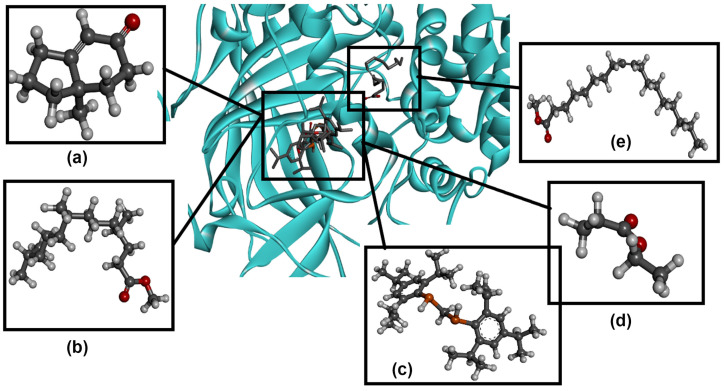
Docking pose scores of the studied ligands (**a**) MHI, (**b**) MTTD, (**c**) MTP, (**d**) PAE and (**e**) OAM with target protein 8CAT.

**Figure 9:**
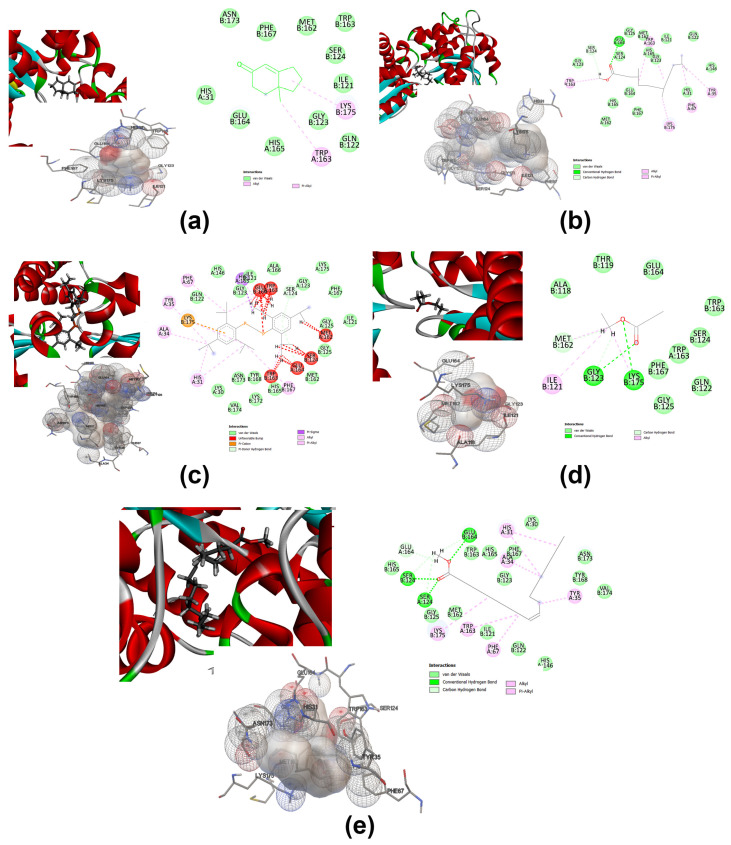
Several non-covalent interactions between the amino acids of 1AR5 and (**a**) MHI, (**b**) MTTP, (**c**) MTP, (**d**) PAE and (**e**) OAM.

**Figure 10:**
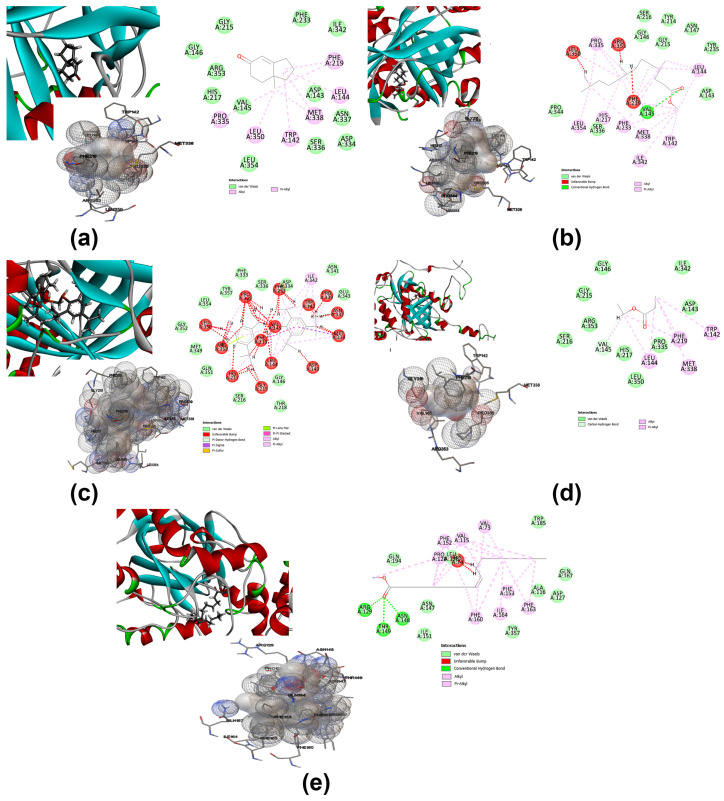
Several non-covalent interactions between the amino acids of 1AR5 and (**a**) MHI, (**b**) MTTP, (**c**) MTP, (**d**) PAE mand (**e**) OAM.

To rationalize the previous experimental activities as antioxidant behaviors, molecular docking data analysis predicted the inhibition of protein active sites. The best conformers and protein–ligand binding states generated from docking are ranked with higher (with negative values) binding energies. Table 2 presents the ligands with binding energy (E_binding_) and intermolecular energy (E_Intermol.)_ values, where the more negative values of binding energy explain the stability of the protein–ligand complex. According to the investigated energy parameters, related to binding energy, it was predicted that MHI is more stable (−5.79 kcal/mol) in complexing with the target protein 1AR5, and also forms a more stable complex (−6.03 kcal/mol) with the target protein 8CAT. This may be attributed to the heterocyclic rings that strongly interact with amino acids independent of their number and type of interaction. As regards other intermolecular interactions, MTTD manifested several interaction types with 1AR5 (−8.24 kcal/mol), while in 8CAT, one type of unfavorable interaction destabilized the ligand–protein complex (12.56 kcal/mol). In both target proteins, MTP yielded a highly unstable bio-complex due to the large steric hindrance and unfavorable bumps that were formed. PAE and OAM with 1AR5 showed a similar binding-stabilization effect (−3.79 and −3.73 kcal/mol, respectively), with larger interaction types in only OAM (−8.56 kcal/mol), while for 8CAT, the protein–PAE complex was found to be more stable than OAM. Based on the data resulting from docking with the two target proteins 1AR5 and 8CAT, the five ligands with a successful pose score interacted with several amino acids. Figure 7 and Figure 8 show the best docking scores at the same protein active sites, with some differences in the mode of interaction. Due to the large variety of structural functional bio-species, Figure 9 and Figure 10 show several non-covalent interaction types present between the bio-macromolecule and the studied ligands, such as Van der Waals, alkyl, π-alkyl, conventional hydrogen and carbon–hydrogen bonds, and the π-donor hydrogen bond. Large unfavorable bumps are distributed in the protein–MTP complex (Figure 8c and Figure 10c), and are also slightly present in the 8CAT-MTTD complex (Figure 10b).

### 3.8. SEM Analysis

**Figure 11:**
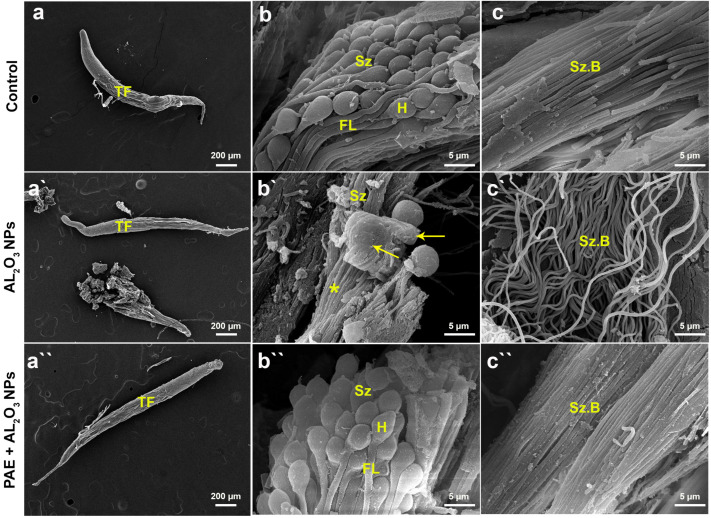
Scanning electron micrographs of the testicular tissues of *L. migratoria* using SEM after Al_2_O_3_ NPs injection with or without PAE treatment. The typical structure of the testicular follicle (TF) from the control group is shown in (**a**) compared to TFs from the Al_2_O_3_ NPs-treated group in = (**a`**) and the PAE + Al_2_O_3_ NPs-treated group in (**a``**). Figures (**b**) and (**b``**) show normal spermatozoa morphology in the control and the PAE + Al_2_O_3_ NPs-treated groups, respectively, with oval heads and long flagella. Figure (**b`**) shows a double-headed spermatid (arrows), and spermatids agglutinated tail-to-tail (*). Figures (**c**–**c``**) show the difference in the regularity and the arrangement of spermatozoa tails within the spermatozoa bundles (Sz. B) in the control in (**c**), the Al_2_O_3_ NPs-treated group in (**c`**), and the PAE + Al_2_O_3_ NPs-treated group in (**c``**), respectively.

Scan electron micrographs of the Al_2_O_3_ NPs-injected locusts reveal anomalies in the morphology of the testicular tissues, with remarkable impairment in the testicular follicle (TF). Additionally, Al_2_O_3_ NPs exposure resulted in significant diminutions in the TF width compared to the control and pre-treated with PAE tissues (Figure 11a, a` and a``). Moreover, abnormalities in spermatozoa (Sz) heads and flagella structures were observed in the Al_2_O_3_ NPs-injected insects compared with the control group and the locusts pre-administered with PAE (Figure 11b–b``). The spermatozoa of locusts exposed only to Al_2_O_3_ NPs emerged with abnormal morphologies, with double-head spermatids and spermatids agglutinated tail-to-tail (Figure 11b`), whereas those from locusts exposed to PAE + Al_2_O_3_ NPs had regular morphological structures and a long flagellum without any abnormalities (Figure 11b``). The spermatozoa bundles in the Al_2_O_3_ NPs-injected locusts appeared with an irregular arrangement compared to the control insects (Figure 11c,c`). It is evident that the spermatozoa bundles in the PAE-treated locusts emerged with regular arrangements similar to those in the control group, and exhibited no signs of agglutinations or structural abnormalities, as illustrated in Figure 11c``. Overall, these findings suggest that PAE may be useful in preventing the testicular structural anomalies caused by Al_2_O_3_ NPs.

### 3.9. Histological Analysis

**Figure 12:**
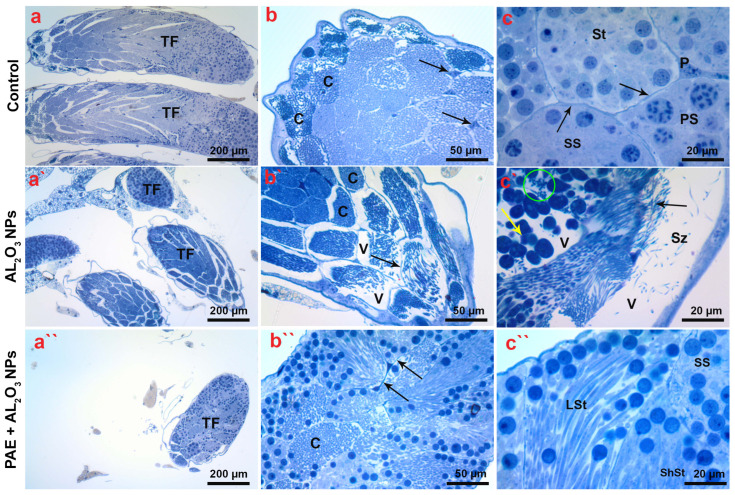
Photomicrographs of semithin sections show the histological structures of the testicular follicles (TF) of the locust after Al_2_O_3_ NPs injection with or without PAE treatment. Semithin sections through the TFs of the control, Al_2_O_3_ NPs, and PAE + Al_2_O_3_ NPs groups are given in (**a**), (**a`**) and (**a``**), respectively, showing shrinkage in the size of follicles in the Al_2_O_3_ NPs- and PAE + Al_2_O_3_ NPs-treated groups compared to the control. Figure (**a**) illustrates the typical structure of the TFs in the control group. Figure (**a`**) shows a shrinkage of follicular size in the Al_2_O_3_ NPs-treated group. Figure (**a``**) exhibits normal TF similar to that of the control group. Figures (**b**–**b``**) show the different cysts (C) within the TFs and parietal cells in between (arrows) in the control, Al_2_O_3_ NPs-, and PAE + Al_2_O_3_ NPs-treated groups, respectively. Figure (**b**) illustrates typical follicular cysts of various spermatogenic stages. Figure (**b`**) shows obvious vacuolation (V) in the cysts of the Al_2_O_3_ NPs-exposed group. Figure (**b``**) demonstrates normal follicular cysts at different developmental stages with intact cyst walls (arrows). Figure (**c**) shows typical spermatogenic elements, indicating different developmental stages, including primary spermatocyte (PS), secondary spermatocyte (SS), and spermatid (St). Besides this, normal parietal cells (P) and cyst walls (arrows) could be perceived. Figure (**c`**) shows the rupture of cyst walls (black arrow), vacuolation (V), dense vesicles (yellow arrow), spermatozoa (Sz) in the follicle lumen, and dense particles (green circle). Figure (**c``**) exhibits regular spermatogenic elements, implying normal developmental stages, including secondary spermatocyte (SS), short spermatid (ShSt), and long spermatid (LSt).

Histological analysis of the TF of locusts treated with Al_2_O_3_ NPs has shown severe morphological and structural aberrations, including remarkable shrinkage in TF size compared to the control insects (Figure 12a–a``). Typical cysts within the TFs and parietal cells in between were discerned in the control and the PAE + Al_2_O_3_ NPs-treated groups. By contrast, ruptured cyst walls and numerous vacuolations within cysts were observed in Al_2_O_3_ NPs-treated locusts, which could not be detected in the controls or the insects treated with PAE + Al_2_O_3_ NPs (Figure 12b–b``).

In the PAE + Al_2_O_3_ NPs locust group, the TF showed various cysts at different developmental stages of the spermatogenic elements, including primary spermatocytes, secondary spermatocytes, and spermatids with typical cyst morphology, intact cyst walls, regular parietal cells, and typical global organization, indicating normal spermiogenesis development (Figure 12c). By contrast, morphologically altered spermatogonia, showing signs of disintegration and necrosis with anomalous staining, dense vesicles, dense particles, and vacuolated cysts without any germ cells, were discernible in Al_2_O_3_ NPs-injected locusts. The severe shrinkage within the TF of Al_2_O_3_ NPs-injected locusts resulted in a distinct separation between the follicular wall and the cyst content, which led to the presence of an empty area throughout the contour of the follicle. This empty area revealed the rupture of the follicular or cyst walls, as depicted in Figure 12c`. Interestingly, the normal organization of the secondary spermatocyte, the short spermatids, and the long spermatids within the cysts was discernible in the locusts pre-exposed to PAE (Figure 12c``).

### 3.10. Ultrastructural Analysis

**Figure 13:**
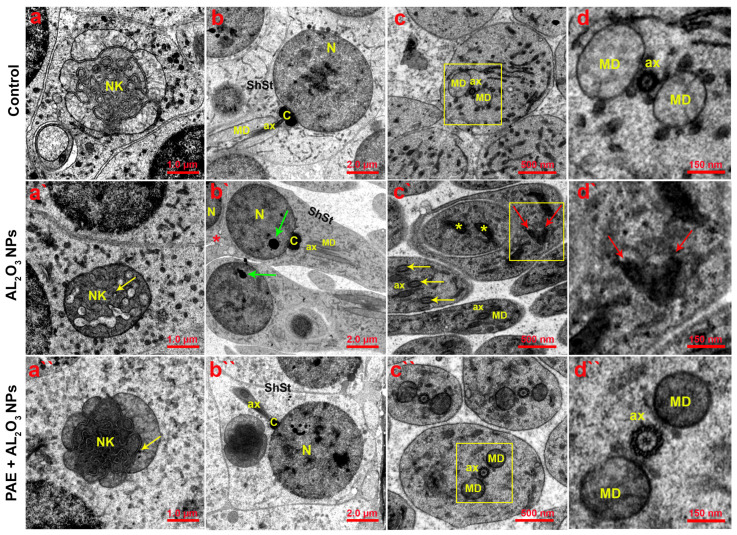
Ultrastructural analysis of the locust testis using TEM after Al_2_O_3_ NPs injection with or without PAE treatment. Electron micrographs in Figures (**a**–**a``**) reveal the difference in the structure of the mitochondrial nebenkern (NK) associated with the early spermatid stage in the control group, the Al_2_O_3_ NPs-treated group, and the PAE + Al_2_O_3_ NPs-treated group, respectively. Figure (**a**) shows the typical structure of NK characteristics for *L. migratoria*. Figure (**a`**) depicts the NK alterations as disintegrated NK with many vacuoles containing nanoparticles (arrow) in locust follicular cysts exposed only to Al_2_O_3_ NPs. Figure (**a``**) demonstrates the normal integration of the NK similar to that of the control group even with the accumulation of Al_2_O_3_ NPs within it (arrow). Electron micrographs in Figures (**b–b``**) show the short spermatid (ShSt) stage in the control group, Al_2_O_3_ NPs-treated group, and PAE + Al_2_O_3_ NPs-treated group, respectively. Figure (**b**) shows the typical structures of the short spermatid (ShSt), nucleus (N), centriole (C), axonemes (ax) and two mitochondrial derivatives (MD). Figure (**b`**) shows a short spermatid (ShSt) agglutinated head-to-head (*), nuclear dense vesicles of aggregated nanoparticles (arrows) (which could not be assessed with TEM analysis), centriole (C), and nucleus (N). Figure (**b``**) shows normal short spermatids (ShSt). Figures (**c**), (**c`**,**c``**) show the structural difference of the flagella as a result of exposure to Al_2_O_3_ NPs with and without the PAE treatment compared to the control group. Figure (**c**) shows a typical transverse section across the flagella, with the axoneme (ax) in the middle, surrounded by two mitochondrial derivatives (MD). Compared to the control group, Figure (**c`**) illustrates the structural anomalies in the flagella, which emerged as degenerated axoneme (ax) (red arrow) and mitochondrial derivatives (MD) (asterisk). Furthermore, agglutination is implied by three axonemes (ax) in the same section (yellow arrow). Figure (**c``**) shows typical axoneme (ax) and mitochondrial derivatives (MD). Figures (**d**–**d``**) depict magnified sections as indicated in the yellow square of the previous figures, exhibiting the structure of normal axonemes (ax) in Figures (**d**,**d``**), while degenerated axonemes (red arrows) could be observed in locusts treated only with Al_2_O_3_ NPs.

To further substantiate the previous findings, we inspected the ultrastructure of the testicular tissues using TEM analysis. The control insects had normal spermatogenic structures, with the mitochondrial nebenkern associated with the early spermatid stage (Figure 13a). However, Al_2_O_3_ NPs-injected locusts showed several aberrations in the spermatogenic elements, including a degenerated nebenkern with vacuoles and Al_2_O_3_ NP accumulation within it (Figure 13a`). Remarkably, despite the presence of a few nanoparticles within the cyst and mitochondrial nebenkern in insects treated with PAE + Al_2_O_3_ NPs, the TF ultrastructure manifested with normal characteristics and no malformations (Figure 13a``).

It could be observed that the short spermatid stage in the control group had a typical structure, as shown in Figure 13b. Conversely, a nuclear dense vesicle of aggregated nanoparticles and agglutinated head-to-head spermatids could be observed in Al_2_O_3_ NPs-exposed group (Figure 13b`). We presume that the black spots observed in this section are likely related to the cluster of nanoparticles, since they were not detected in the control insects, but the exact nature of the spots is not adequately assessable with TEM analysis. On the other hand, a normal short spermatid was perceived in the group pretreated with the PAE (Figure 13b``). Additionally, transversal sections across the flagella revealed a typical axoneme in the middle, surrounded by two mitochondrial derivatives (Figure 13c). On the other hand, locusts exposed only to Al_2_O_3_ NPs emerged with various structural anomalies in the flagella, including a degenerated axoneme and malformed mitochondrial derivatives. Furthermore, agglutination is implied by three axonemes in the same section (Figure 13c`). However, pretreatment with PAE resulted in the emergence of normal flagella with a typical axoneme and mitochondrial derivatives (Figure 13c``). The TEM analysis confirms our previous findings, emphasizing the vital role of PAE as a prophylactic extract in attenuating the toxicity induced by Al_2_O_3_ NPs.

## 4. Discussion

Recently, a considerable amount of literature has proliferated around the importance and broad applications of aluminum nanoparticles. In light of this event, it is becoming extremely problematic to disregard the incidence and bioaccumulation of these nanoparticles in living organisms. Thus, several authors have attempted to investigate the deleterious influences of Al NPs on mammals and in vitro using cell lines. They reported that Al NPs can induce oxidative stress in different types of cells, which could further engender adverse consequences for the metabolic pathways and the functions of several organs [10,37]. However, no reports have comprehensively studied the impacts of Al NPs on testicular tissues of migratory locusts. On the other hand, previous investigations sought to discover antioxidant agents derived from plants, particularly those containing polyphenolic compounds, to curtail the toxicity of the nanoparticles and treat the oxidative testicular injury triggered by their indirect influence on cells existing in testes [18,19]. However, the antioxidant compounds extracted from the insects have received scant attention in empirical research. In this work, we extensively studied the influence of Al_2_O_3_ NPs on the testicular tissues of *L. migratoria* as an insect model and sought to suppress their harmful effects through the pre-treatment of locusts with PAE as a prophylactic agent.

### 4.1. Size and Bioaccumulation of Al_2_O_3_ in the Testicular Tissues

Admittedly, the size of nanoparticles makes a decisive contribution to the degree of their harmfulness, since it affects their infiltration throughout the tissues [3]. The SEM and TEM analyses revealed that Al_2_O_3_ NPs have an average size of 62 nm, indicating the facile translocation of the nanoparticles throughout the locust’s tissues.

EDX examination of the testicular tissues harvested from the control group of locusts revealed the incidence of C, N, O, Na, P, and S, while the locust group exposed to Al_2_O_3_ NPs demonstrated the agglomeration of Al in addition to the previous elements. The pretreatment of locusts with PAE resulted in a significant lessening of the Al accumulated in the testes. This is likely related to the chelating activity of PAE, due to the presence of several compounds with high antioxidant features along with chelation properties in relation to the nanoparticles [20].

### 4.2. Impact of Alumina and PAE on Hemocytes

Hemocytes are predominantly assumed to play a crucial immune role in animals with regard to cellular and humoral responses [38]. Thus, an increase in hemocytes is likely stimulated to promote the detoxification process of pollutants and other toxic substances [39]. Our findings have demonstrated that a single administration of Al_2_O_3_ NPs resulted in hemocyte activation and an increase in THC. Previous studies revealed that exposure to CuO NPs and ZnO NPs increased THC levels in the hemolymph of *Galleria mellonella* and *Bombyx mori*, respectively [40,41]. In accordance with our findings, studies on *G. mellonella* have suggested that this growth may be attributed to the enhancement of hematopoiesis or the immune response on account of the activation of the mitotic division of hemocytes in response to the accumulation of Al NPs [39,40]. In contrast to these findings, the THC was lessened as a result of pre-administration with PAE, implying the capacity of the extract to govern the inflammation provoked by Al_2_O_3_ NPs.

### 4.3. Al-induced Oxidative Stress in the Locust Testes

In the current study, in the testicular tissues from the Al_2_O_3_ NPs group, we reported augmentations of MDA, SOD, and CAT levels, whereas the activities of TAC, GPx, CarE, and GST were substantially decreased. ErbaŞ and AltuntaŞ [42] proposed that the increase in CAT and SOD activities in *G. mellonella* could be attributed to an adaptive response to the oxidative damage provoked by xenobiotics. In the same manner, we suggest that the increased levels of CAT and SOD in the Al_2_O_3_ NPs may arise as a spontaneous metabolic reaction to acclimatize to the agglomeration of Al inside the testicular tissues.

Furthermore, the MDA level was heightened in the testes as a result of exposure to a single dose of Al_2_O_3_ NPs, implying high lipid peroxidation. It is worth mentioning that previous investigations demonstrated that GPx functions to enhance the detoxification of lipid hydroperoxides, which stem from lipid peroxidation (LPO), and it could even hamper the instigation of LPO [42]. Therefore, the decreased levels of the antioxidant enzymes (TAC and GPx) in the testicular tissues could be explained by a rise in LPO activity that may be related to the reduction in antioxidant enzymes implicated in the detoxification of ROS in these tissues, to sustain the antioxidant defense system. By contrast, the pretreatment of locusts with PAE promoted the performances of TAC and GPx, which explains the antioxidant characteristics of the extract. In addition, the remarkable inhibition of CarE and GST activities in the Al_2_O_3_ NPs-treated group indicates their deficiency in repelling the oxidative stress, which further gives rise to the impairment of the testicular tissues. However, the amplification of TAC capacity in the PAE-treated group discloses that the antioxidant defense system was immensely active in modulating the oxidative stress derived from the discharge of ROS. Therefore, the improved enzymatic activities in the PAE treated animals were probably due to the protective role of PAE against Al_2_O_3_ NPs-mediated toxicity.

It is being recognized that metabolic enzymes play a vital role in preventing the oxidative damage resulting from xenobiotic agents. Thus, it was reported in the locusts exposed to Al_2_O_3_ NPs that the activities of ALT and AST were significantly enhanced, while the protein contents were markedly diminished. These findings are in agreement with those observed in the ground beetle as a result of soil pollution with heavy metals [43,44]. Previous studies have suggested that a decrease in total protein could be considered a strong sign of the dysregulation of enzyme activities and expressions, as elaborated above [45]. It is well known that heat shock proteins (HSPs) play a paramount role in insects and mammals to maintain cellular homeostasis [46]. In our previous work, we found a noticeable upregulation of specific protective proteins, such as HSPs, as a defense strategy to govern oxidative stress [47]. Our findings demonstrate that the expressions of HSP70 and HSP90 were highly upregulated in the Al_2_O_3_ NPs-exposed animals compared to the controls. However, significant inhibition of their expression was observed in the insects pretreated with PAE. Previous studies reported an increase in HSP70 levels in *A. domesticus* after exposure to nanodiamonds [48] and to nanographene oxide [49]. On the other hand, the increase in the NO level in the locusts treated only with Al_2_O_3_ NPs clearly indicates the unfavorable stress inside the cells, which was significantly abated in the group treated with PAE. Prior studies have posited that nitric oxide has the ability to react with superoxide, producing peroxynitrite. Given the intense oxidative capacity of peroxynitrite, it could interact with the molecules in the biological system, which further thwarts the activity and functions of imperative metabolic enzymes, disrupting the integrity of mitochondria [19,50]. Nevertheless, the remarkable diminution in the NO in locusts pretreated with PAE points to the capacity of the antioxidant compounds in PAE to scavenge the NO, impeding the other adverse effects. To explain the deleterious consequences of Al_2_O_3_ NPs on the basis of these findings, we presume that the interaction of these nanoparticles is comparable to that of other counterparts, such as AgNPs [6,7]. Thus, we could postulate that the toxicity of Al_2_O_3_ NPs could be instigated through the surface oxidation of the nanoparticles by oxygen and other oxidized agents, which predominate in living organisms, stimulating the discharge of free Al inside the cells. This could result in an excess of ROS, which could lead to other severe complications, such as inflammation and cell cycle disorders.

Surplus ROS could impair the integrity of the lipid membrane and augment membrane permeability [51]. This leads to protein impairment, including modifications of amino acids, the disintegration of peptide chains, critical aggregation, and uncontrolled cross-linking reactions, and indeed, these cause the considerable production of non-functionalized proteins and enzymatic deactivation [51,52].

From these findings, it could be deduced that the toxicity of Al_2_O_3_ NPs could result in the dysfunction of the reproductive system by inciting oxidative injury in the testicular tissues. On the other hand, the pre-administration with PAE efficiently attenuated the direct and indirect deleterious effects of Al_2_O_3_ NPs through the amelioration of the antioxidant defense mechanism, which further impedes oxidative stress. Based on recent studies, the antioxidant activity of PAE may be mainly attributed to the existence of various antioxidant compounds in the PAE, since the authors have demonstrated the presence of sixty compounds with different structures and properties, such as dopamine, coumarin, aminoacids, dipeptide, and organic compounds, which make crucial contributions to controlling the overproduction of ROS, leading to an improvement of the antioxidant enzymes and other oxidative parameters [20,23]. Overall, the molecular docking analysis and the in vivo studies emphasized the antioxidant competency of the PAE.

### 4.4. Apoptotic Analysis and DNA Damage of Testicular Tissues

It is assumed that oxidative stress caused by an overflow of ROS disrupts the antioxidant metabolic system of the cells, provoking apoptosis, which is predominantly associated with DNA damage. Moreover, the unregulated ROS impair the DNA by oxidizing deoxyribose, fragmenting the DNA strand, altering bases, and further leading to critical mutations in the expressed proteins [51,53,54]. On the other hand, previous studies postulated an alternative interaction of nanoparticles, such as Ag and Al, with DNA that depends on the infiltration of nanoparticles into the nucleus through nuclear pores and binding to the DNA of the cells, modifying the structure of the DNA and leading to either DNA damage or unanticipated mutations [7,55]. Furthermore, the integrity and functions of mitochondria are prone to critical disorders due to the overproduction of ROS, which is usually modulated by the antioxidant mechanism of mitochondria. However, the accumulation of Al results in an overabundance of ROS, causing oxidative stress associated with a disturbance of ATP synthesis. Accordingly, the anticipated fate of cells triggered by these critical dysregulations includes DNA injury and apoptosis. According to our data, Al_2_O_3_ NPs induced DNA impairment, which is consistent with previous reports that showed an increase in DNA damage in *A. domesticus* after the administration of nanodiamond [48] and graphene oxide [55]. Although the Al_2_O_3_ NPs accumulated within the testicular tissues, the group pretreated with PAE showed a reduction in DNA damage compared to the group treated only with Al_2_O_3_ NPs. Consequently, our results demonstrate that PAE can protect DNA from the genotoxic effect of alumina NPs. Regarding the health status of locust cells in our study, several investigations have reported an elevation in apoptotic and necrotic cells due to metal NPs’ toxicity [7,56]. Importantly, the PAE-treated group exhibited improved cell survival, as a higher percentage of viable cells combined with reduced percentages of apoptotic and necrotic cells were observed. However, the complete restoration of the cell’s viability requires a longer time after eradicating the major deleterious influences of Al_2_O_3_ NPs. Altogether, the pre-treatment of locusts with PAE may protect cells from the negative effects of Al_2_O_3_ NPs by suppressing DNA damage and inhibiting cell apoptosis. 

### 4.5. Histological and Ultrastructural Analyses of Testicular Tissues

To provide more evidence on the protective influence of PAE in relation to attenuating the hazardous episodes provoked by the toxicity of the testicular tissues with Al_2_O_3_ NPs, we inspected the histological and ultrastructural characteristics of the testes compared to the testicular tissues exposed to a single application of Al_2_O_3_ NPs and those from control locusts. Prior investigations have evidenced that treatment with either Ag NPs or NiO NPs incited various anomalies in the testicular tissue of beetles, including deformed spermatogenic elements and agglutinated spermatids, in addition to axonemal and mitochondrial deformations [4,47,57]. Furthermore, Wang et al. [58] reported that exposure to nanoparticles such as TiO_2_, gold alloys, nickel NPs, and Ag NPs had serious effects on the male reproductive system. In this study, histological, SEM, and TEM analyses demonstrated different aberrations in testicular tissues from locusts treated with Al_2_O_3_ NPs, particularly a substantial degradation of the mitochondria. The disintegration of mitochondrial structures and their derivatives conceivably disrupts the ATP supply for sperm motility, leading to infertility [4,47]. These observations imply the alarming toxicity of Al_2_O_3_ NPs connected with bioburden toward cells, even if they are administered in low doses. Kheirallah et al. [47] elucidated that the secondary fusion of the spermatogenic elements during mitosis could be the underlying cause behind the appearance of bi- and tetra-flagellated sperms. In contrast to these findings, the testicular tissues of the group pretreated with PAE showed the ameliorative capacity of PAE and its role in protecting testicular tissues, with no manifestations of sperm cell agglutination. Given this fact, the treatment of locusts with PAE could sustain the integrity of sperms against the adverse effects of Al_2_O_3_ NPs, and we could therefore presume that the behavior of sperms would return to normal with regard to motility and the fertilization process accordingly. In a similar fashion, different plant extracts, such as curcumin [59] and *Aloe vera* gel [60], exhibited ameliorative and protective effects against the Al- and AlCl_3_-induced reproductive toxicity of Wistar rats. This research raised many questions in need of further investigation. Despite the widespread application of PAE in traditional Chinese medicine [61] and previous studies demonstrating its safe and effective application to accelerate diabetic wound healing [62,63], the mortality test of adult locusts using different concentrations of PAE revealed a mortality rate of 20%. Therefore, further investigations are required to understand the underlying mechanisms and interactions of PAE within cells of different organs. Future studies on the purification of the PAE compounds are therefore recommended, since it would be interesting to ascertain the compounds with high antioxidant and anti-inflammatory capacities. Moreover, the antagonistic and synergistic effects of the compounds could be evaluated to explore the potential candidates for promoting their biomedical applications while minimizing their side effects. Notwithstanding these limitations, our findings substantiate the prophylactic effect of PAE against Al_2_O_3_ NPs-induced reproductive toxicity in the testicular tissue of the locust model as an animal model, maintaining the structure and function of the testicular tissues. These findings emphasize the results obtained from the biochemical, DNA damage, and apoptotic analyses.

## 5. Conclusions

To sum up, our work offers comprehensive insights into the deleterious effects instigated by exposure to Al_2_O_3_ NPs at a low dose in the testicular tissues of *L. migratoria*, along with the extent of the influence of *P. americana* extract (PAE) as a prophylactic. Our results demonstrate that the release of Al as a result of Al_2_O_3_ NPs oxidation caused disruption of the antioxidant defense system in the testes, resulting in DNA injury, cells apoptosis and alterations in mitochondria and other organelles in the testicular tissue. In contrast, pretreatment with PAE precluded all the harmful effects of Al accumulation at all levels, including the activity of enzyme and non-enzymatic antioxidant factors, the expression of stress biomarkers, DNA impairment, and cell apoptosis. Besides this, no anomalies were perceived in the histological and ultrastructural sections of testicular tissues pretreated with PAE. These findings suggest the potential implementation of PAE to attenuate the oxidative stress and other consequences originating from the toxicity of metal NPs toward the male reproductive system.

## Figures and Tables

**Table 1:** EDX analysis of testicular tissues from *L. migratoria* in response to Al_2_O_3_ NPs and PAE treatment.

Control Group			Group Treated with Al_2_O_3_ NPs		Group Treated with PAE + Al_2_O_3_ NPs	
Element	Mass (%)	Atom (%)	Mass (%)	Atom (%)	Mass (%)	Atom (%)
C	55.47 ± 0.30	62.00 ± 0.33	43.97 ± 0.29	50.12 ± 0.33	44.86 ± 0.29	51.10 ± 0.33
N	9.63 ± 0.56	9.23 ± 0.54	18.83 ± 0.66	18.40 ± 0.64	20.93 ± 0.66	20.45 ± 0.65
O	33.45 ± 0.61	28.07 ± 0.52	36.26 ± 0.70	31.03 ± 0.60	32.11 ± 0.65	27.46 ± 0.56
Na	0.46 ± 0.06	0.27 ± 0.03	0.13 ± 0.05	0.08 ± 0.03	0.46 ± 0.06	0.28 ± 0.03
P	0.63 ± 0.04	0.27 ± 0.02	0.24 ± 0.04	0.11 ± 0.02	0.74 ± 0.05	0.33 ± 0.02
S	0.36 ± 0.03	0.15 ± 0.01	0.33 ± 0.04	0.14 ± 0.02	0.83 ± 0.05	0.35 ± 0.02
Al	---	---	0.25 ± 0.04	0.13 ± 0.02	0.07 ± 0.03	0.03 ± 0.01

Data are presented as mean ± SD as a result of investigations of three tissues dissected from three insects from each group.

**Table 2:** Energy parameter values from the molecular docking of the studied ligands against the oxidant activity of 1AR5 and 8CAT.

Ligand	1AR5		8CAT	
	E_binding_ (kcal/mol)	E_Intermol._ (kcal/mol)	E_binding_ (kcal/mol)	E_Intermol._ (kcal/mol)
MHI	−5.79	−6.09	−6.03	−6.93
MTTD	−4.66	−8.24	16.14	12.56
MTP	171.76	168.78	470.36	467.37
PAE	−3.79	−4.68	−4.12	−5.02
OAM	−3.73	−8.56	20.11	15.34

## Data Availability

The datasets generated during the current study are available from the corresponding authors upon reasonable request.
